# Yiqi Fumai Injection as an Adjuvant Therapy in Treating Chronic Heart Failure: A Meta-Analysis of 33 Randomized Controlled Trials

**DOI:** 10.1155/2020/1876080

**Published:** 2020-08-19

**Authors:** Heyun Nie, Shuqing Li, Meilu Liu, Weifeng Zhu, Xu Zhou, Dongmei Yan

**Affiliations:** Evidence-based Medicine Research Center, Jiangxi University of Traditional Chinese Medicine, Nanchang, Jiangxi, China

## Abstract

**Background:**

Yiqi Fumai injection (YQFM) is a traditional Chinese medicine widely used for cardiovascular diseases in China. This systematic review aimed to evaluate whether YQFM could be an effective and safe complementary therapy for chronic heart failure (CHF).

**Methods:**

Eight electronic literature databases were searched up to March 31, 2020. Randomized controlled trials (RCTs) comparing YQFM + conventional treatment with conventional treatment alone for CHF were included. The primary outcome was response to treatment, which was graded by improvements in heart function based on the New York Heart Association (NYHA) criteria, while the secondary outcomes included the left ventricular ejection fraction (LVEF), cardiac output, left ventricular end-systolic diameter (LVESD), amino-terminal pro-brain natriuretic peptide (NT-proBNP), 6-minute walk test performance (6MWT), quality of life (QoL) as assessed by the Minnesota Living with Heart Failure questionnaire, and adverse reactions. Data from individual RCTs were pooled by a random-effects meta-analysis with effect measures of proportional odds ratios (pORs) and 95% confidence intervals (95% CIs) for the ordinal outcomes and the mean difference (MD) and 95% CI for the continuous outcomes.

**Results:**

In total, 33 RCTs involving 3070 patients with an overall moderate-to-high risk of bias were selected. The meta-analysis showed that compared with conventional treatment alone, YQFM plus conventional treatment had a significantly higher likelihood of improving the response to treatment (pOR 1.88, 95% CI 1.47 to 2.42, *I*^2^ = 0%). YQFM also significantly improved the LVEF (MD 5.53%, 95% CI 4.73 to 6.33, *I*^2^ = 82%), cardiac output (MD 0.32 L/min, 95% CI 0.19 to 0.45, *I*^2^ = 47%), and LVESD (MD −3.73 mm, 95% CI −5.51 to −1.95, *I*^2^ = 22%), reduced the NT-proBNP levels (MD −341.83 pg/mL, 95% CI −417.89 to −265.77, *I*^2^ = 88%), and improved the 6MWT (MD 61.86 m, 95% CI 45.05 to 78.67, *I*^2^ = 64%) and QoL (MD −9.82, 95% CI −14.17 to −5.46, *I*^2^ = 81%). No serious adverse events related to YQFM were reported.

**Conclusion:**

Although limited by a moderate-to-high risk of bias, the current evidence suggests that YQFM as a complementary treatment significantly improves heart function and related indicators in patients with CHF. The clinical use of YQFM needs careful safety monitoring. Well-designed studies are still required to further evaluate the efficacy and safety of YQFM for CHF.

## 1. Introduction

Chronic heart failure (CHF) is a major cardiovascular disease that manifests as myocardial structural and functional damage and persistent left ventricular systolic dysfunction [1]. With the ageing of the population and changes in people's lifestyle, the incidences of the primary diseases of CHF, such as hypertension, diabetes, and coronary heart disease, are rapidly increasing, and the global burden of CHF is rising annually. In the United States, the annual incidence of CHF among people aged over 65 is 19.3 per 1,000 people, and the annual direct and indirect costs of treating CHF exceed 39 billion dollars [2]. In 2015, the prevalence of CHF in the Chinese population aged over 65 years reached 10% [3]. CHF patients have a poor prognosis and a high mortality rate. The one-year hospitalization rates of inpatients and stable CHF patients were 44% and 32%, respectively [4]. The five-year mortality rate of CHF is as high as 60%–80%, which is similar to that of malignant tumours, such as breast cancer and colorectal cancer [3]. Sudden cardiac death, which is the main cause of death (40.2%), is difficult to prevent and rescue [5].

Currently, the drugs recommended in clinical practice guidelines for CHF mainly include diuretics, angiotensin-converting enzyme inhibitor, *β*-receptor blocker, aldosterone receptor antagonists, and digitalis [1], all of which are limited by contraindications and adverse effects. For example, diuretics can result in hypovolemia associated with hypotension, renal function deterioration, and electrolyte imbalance [4]; digoxin can easily cause poisoning manifested as gastrointestinal adverse reactions, visual disturbances, and arrhythmias [6]. In addition, these drugs are expensive, causing a tremendous economic burden for patients and medical care systems. It is expected that, by 2030, the annual total medical expenses for CHF patients could increase to $53 billion [7]. Therefore, there is a need for more CHF treatment choices.

In China, traditional Chinese medicine (TCM) as a complementary therapy has been widely used for cardiovascular diseases. In particular, innovative TCM injections, such as Yiqi Fumai injections (YQFM), provide new options for CHF treatment. The compounds of YQFM include the active ingredients of the following three herbs: total saponins panax ginseng from red ginseng, ophiopogonin from Radix Ophiopogonis, and schizandrol from *Schisandra chinensis*; these compounds are finally prepared in a freeze-dried powder injection. This preparation can overcome the storage and transportation inconveniences related to the instability of TCM injections [8]. YQFM can be easily dissolved in normal saline for an intravenous drip and has the advantages of rapid action, a high concentration of active ingredients, and accurate dosing. Pharmacological studies have demonstrated the cardioprotective effects of YQFM, including reducing myocardial ischaemia and hypoxia injury, enhancing the systolic function of the heart, delaying ventricular remodelling, inhibiting cardiomyocyte apoptosis, and ultimately improving cardiac function [9].

Since its approval for marketing in 2007, the efficacy and safety of YQFM for CHF have been assessed in many RCTs. However, the results are inconsistent likely due to the insufficient sample sizes. For example, Zhang [10] and Yu et al. [11] found that YQFM injection significantly improved both the response to treatment and left ventricular ejection fraction (LVEF) in patients with CHF, but the findings reported in the RCTs of Zhai and Hui [12] and Xue et al. [13] suggested negative results. Therefore, we systematically reviewed currently available RCTs of YQFM as a complementary therapy for CHF and aimed to provide more compelling evidence by pooling individual RCT data using meta-analytic methods.

## 2. Methods

The reporting of this study was guided by the Preferred Reporting Items for Systematic Reviews and Meta-analyses checklist.

### 2.1. Eligibility Criteria

Eligible RCTs compared YQFM + conventional treatment with the same conventional treatment alone for the treatment of patients with CHF and reported any outcomes of interest. Patients should be diagnosed with CHF by any recognized criteria such as the New York Heart Association (NYHA) criteria, the Chinese Medical Association criteria, the Framingham criteria, or the World Health Organization criteria, with no limitations on age, sex, and disease course. Studies enrolling patients with other heart diseases, such as acute heart failure, obstructive cardiomyopathy, hypertrophic cardiomyopathy, and atrial fibrillation, were excluded. Eligible conventional treatment could include diuretics, angiotensin-converting enzyme inhibitor, *β*-receptor blocker, digitalis preparation, and aldosterone receptor antagonists. Other TCM preparations and ingredients that were the same as those found in YQFM were not allowed in either the YQFM group or the control group.

### 2.2. Literature Search

Four Chinese databases (Chinese National Knowledge Infrastructure, Wanfang Data, CQVIP, and the Chinese Biomedical Literature Database) and four English databases (PubMed, EMBASE, the Cochrane Library, and Clinicaltrials.gov) were searched. The keywords used in the search included “Yiqi Fumai,” “heart failure,” and “cardiac insufficiency.” For example, the search strategy for PubMed is as follows: (Yiqifumai[tw] OR Yiqi Fumai[tw] OR Yi qi Fu mai[tw]) AND (heart failure[mh] OR heart failure[tw] OR heart insufficiency[tw] OR heart decompensation[tw] OR cardiac failure[tw] OR cardiac insufficiency[tw] OR cardiac decompensation[tw]). Additionally, the major cardiovascular journals and the references of the included studies and related reviews were manually searched. The search was conducted from the inception of each database to March 31, 2020. There was no limitation on the publication language.

### 2.3. Literature Screening

Two reviewers performed an independent, repeated screening of the retrieved papers. The titles and abstracts were first read to exclude studies that did not meet the inclusion criteria, and then, the full text was read and rescreened to determine the RCTs that would be finally included. Disputes were settled through discussion with a third researcher.

### 2.4. Data Extraction

Two reviewers independently and repeatedly extracted the data from the included literature and cross-checked them. Disputes were settled through discussion with a third researcher. The extracted data included the baseline characteristics and outcomes of interest of each study. The former included the first author's name, publication year, sample size, patient characteristics, diagnostic criteria, NYHA class of cardiac function, interventional and control measures, dose and course of treatments, and length of follow-up. The latter included the number or percentage of events, mean, and standard deviation for analysis.

### 2.5. Outcomes

#### 2.5.1. Primary Outcome

The primary outcome was response to treatment, which was categorized into the following three groups according to the NYHA cardiac function classification: (1) marked response: achievement of class I heart function or improvement in heart function by more than two classes; (2) moderate response: improvement in heart function by one class; and (3) no response: no improvement or deterioration in heart function.

#### 2.5.2. Secondary Outcomes

The secondary outcomes included the LVEF, cardiac output, left ventricular end-systolic diameter (LVESD), N-terminal pro-brain natriuretic peptide (NT-proBNP), 6-minute walk test (6MWT), quality of life (QoL) as assessed by the Minnesota Living with Heart Failure questionnaire, and adverse events (AEs).

### 2.6. Risk of Bias Assessment

The Cochrane risk of bias assessment tool was used to assess the level of the risk of bias for each included RCT, which was assessed for seven items: random assignment methods, allocation concealment, investigator and patient blindness, blind evaluation of outcomes, data completeness, selective reporting, and other sources of bias risk. Each item was rated as “low risk,” “unclear,” or “high risk”. We finally judged the overall risk of bias of each RCT based on the following criteria [14]: (1) overall low risk of bias: no items suffer a risk of bias; (2) overall moderate risk of bias: 1–3 items have an unclear risk of bias; and (3) overall high risk of bias: ≥1 item suffer a high risk of bias or ≥4 items have an unclear risk of bias. Two reviewers working independently performed the assessments. Disputes were settled through discussion with a third researcher.

### 2.7. Statistical Analysis

The meta-analysis was performed using Review Manager 5.3. Mean differences (MDs) were used as the effect measures for continuous variables, and proportional odds ratios (pORs) were used as the effect measures for ordinal variables and their 95% confidence intervals (CIs) were also calculated. In this meta-analysis, data from the individual studies were pooled by the inverse variance method with a random-effects model. Heterogeneity was assessed using the Chi-square test and I^2^ statistic; *p* > 0.1 or *I*^2^ ≤ 50% were considered indicative of nonsignificant heterogeneity among studies, and other values were considered indicative of significant heterogeneity. Additionally, subgroup analyses stratified by the daily dose of YQFM (low dose: ≤2.6 g; medium dose: 3.9 g; and high dose: ≥5.2 g), patients' average age (<60 vs ≥ 60 years), and level of overall risk of bias (moderate vs. high) were performed to explore the source of heterogeneity. If, at least, 10 studies were included in the meta-analysis, funnel plots, Egger's test, and Begg's test were used to evaluate publication bias.

## 3. Results

### 3.1. Screening Results

**Figure 1:**
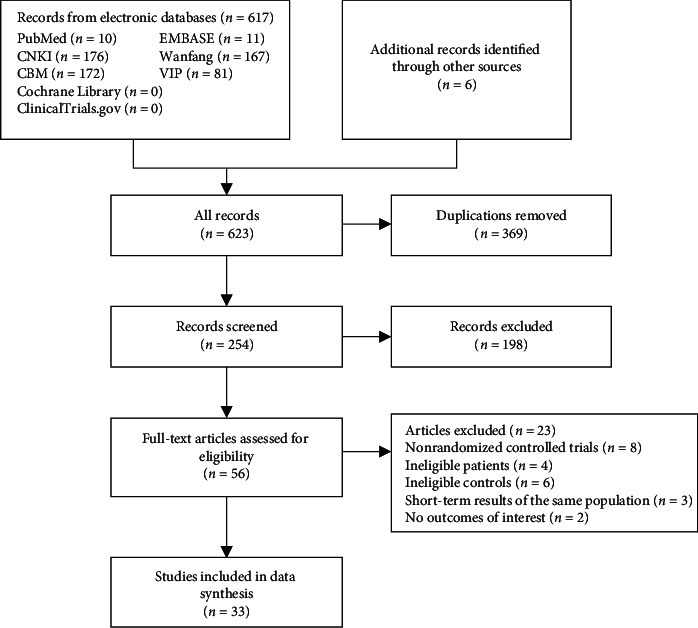
Flowchart of the selection process.

A total of 617 publications were obtained in the initial screening. After reading the titles, abstracts, and full texts, 33 RCTs [10–13, 15–43] with a total of 3070 patients were eventually included. All RCTs were from China. The selection process is shown in Figure 1.

### 3.2. Characteristics of the Included RCTs

Among the 3070 patients, the male to female ratio was approximately 3 : 2. The course of the YQFM treatment was two weeks or less in most RCTs (31, 93.9%); only two RCTs had a four-week course of treatment. The daily dose of YQFM was ≤2.6 g in nine (27.3%) RCTs; 3.9 g in five (15.2%) RCTs; and ≥5.2 g in 18 (54.5%) RCTs. Conventional treatment mainly included diuretics (furosemide, spironolactone), angiotensin-converting enzyme inhibitors (ramipril, captopril, and enalapril), *β*-blockers (metoprolol), and digitalis preparation (digoxin). The baseline NYHA class of the CHF patients was reported in 20 RCTs, of which a total of 336 (23.0%) patients were class II, 852 (58.3%) were class III, and 274 (18.7%) were class IV. The length of the follow-up was the same as the course of treatment in all RCTs. The baseline characteristics are presented in detail in Table 1.

### 3.3. Risk of Bias Assessment

The detailed random sequence generation method was reported in 13 of the RCTs, of which six used the random number table method and one used the random lottery method (low risk), but six used an inappropriate distribution method such as order of admission (high risk). The allocation concealment method was not mentioned in any of the RCTs. None of the RCTs mentioned the blinding method for patients and clinicians (unclear risk), except for one RCT [36], which claimed that a random single-blinded method was used, which was also rated as “high risk” because there was no placebo group to allow blinding. Although none of the RCTs mentioned whether the outcome assessors were blinded, the RCTs with no subjective outcomes were considered to have a low risk of bias for this item. Three RCTs lacked data and did not mention the reasons for loss to follow-up, and thus, the item “data completeness” was rated as “high risk” for those studies. Eight RCTs did not report the important outcomes for CHF, and thus, the item “selective reporting” was rated as “high risk”. Overall, 17 (51.5%) RCTs were judged to have a moderate risk of bias, 16 (48.5%) RCTs had a high risk of bias, and no RCTs were at a low risk of bias. The details of the risk of bias assessment are shown in Figures S1-S2 in the supplementary files.

### 3.4. Evaluation of Outcomes

#### 3.4.1. Response to Treatment

**Figure 2:**
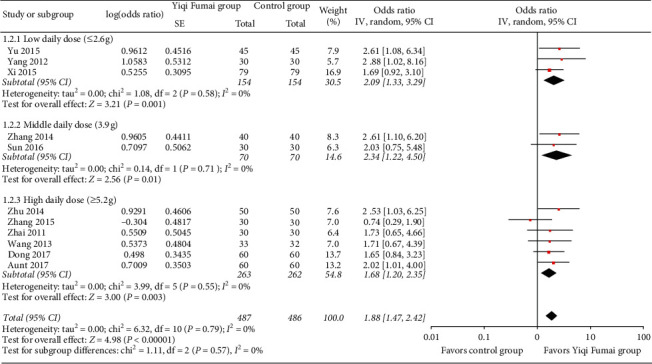
Meta-analysis of the response to treatment.

Eleven RCTs (*n* = 969) [10–12, 15, 17, 27, 31, 35, 39, 41, 43] evaluated response to treatment. After treatment, 194 (39.8%), 245 (50.3%), and 48 (9.9%) patients in the YQFM group and 130 (27.0%), 248 (51.4%), and 104 (21.6%) patients in the control group had a significant, moderate, and no response to treatment, respectively. As Figure 2 indicates, the possibility of improving by more than one class of clinical efficacy was significantly higher in the YQFM group than in the control group (pOR 1.88, 95% CI 1.47 to 2.42, *p* < 0.00001), with no significant statistical heterogeneity (*I*^2^ = 0%; *p*=0.79) among the RCTs.

#### 3.4.2. LVEF

**Figure 3:**
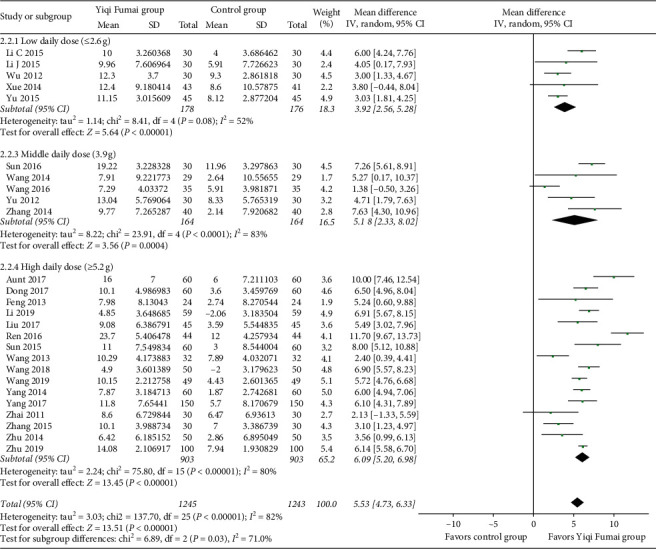
Meta-analysis of the left ventricular ejection fraction (%).

Twenty-six RCTs (*n* = 2488) [10–13, 15, 17–19, 21–23, 26–34, 36, 37, 40–43] reported LVEF data. The random-effects meta-analysis found that the increase in LVEF in the YQFM group was significantly higher than that in the control group (MD 5.53%, 95% CI 4.73 to 6.33, *p* < 0.00001; Figure 3). Heterogeneity was high (*I*^2^ = 82%).

#### 3.4.3. Cardiac Output

**Figure 4:**
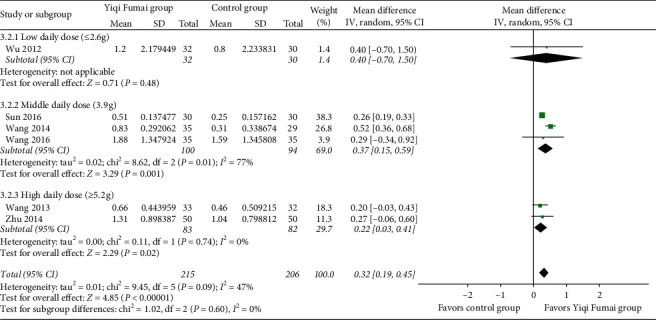
Meta-analysis of the cardiac output (L/min).

Six RCTs (*n* = 421) [27, 29, 31, 33, 34, 43] evaluated the cardiac output of CHF patients. Cardiac output relative to baseline improved significantly more in the YQFM group than in the control group (MD 0.32 L/min, 95% CI 0.19 to 0.45, *p* < 0.00001; Figure 4), with relatively low statistical heterogeneity among the RCTs (*I*^2^ = 47%).

#### 3.4.4. LVESD

**Figure 5:**
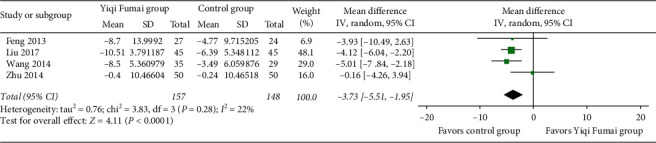
Meta-analysis of the left ventricular end-systolic diameter (mm).

Four RCTs (*n* = 305) [18, 23, 29, 43] reported LVESD data. The YQFM group had a higher reduction in LVESD than the control group (MD -3.73 mm, 95% CI −5.51 to −1.95, *p* < 0.00001; *I*^2^ = 22%; Figure 5).

#### 3.4.5. NT-proBNP

**Figure 6:**
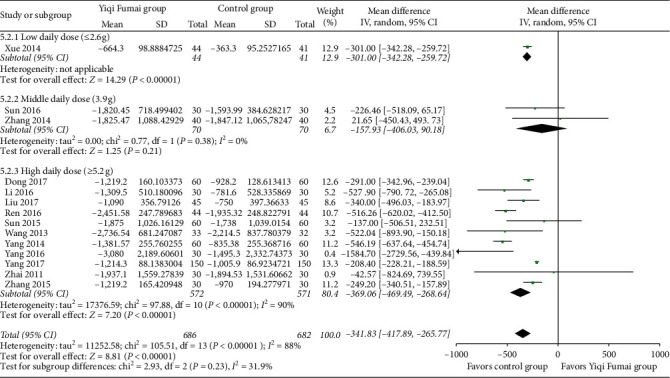
Meta-analysis of N-terminal pro-brain natriuretic peptide (pg/mL).

Fourteen RCTs (*n* = 1368) [10, 12, 13, 17, 20, 23, 26–28, 31, 36–38, 41] reported the evaluation results of NT-proBNP. The YQFM group had a significantly greater reduction in NT-proBNP than the control group (MD −341.83 pg/mL, 95% CI −417.89 to −265.77, *p* < 0.00001; Figure 6), and there was considerable heterogeneity (*I*^2^ = 88%) among the RCTs.

#### 3.4.6. 6MWT

**Figure 7:**
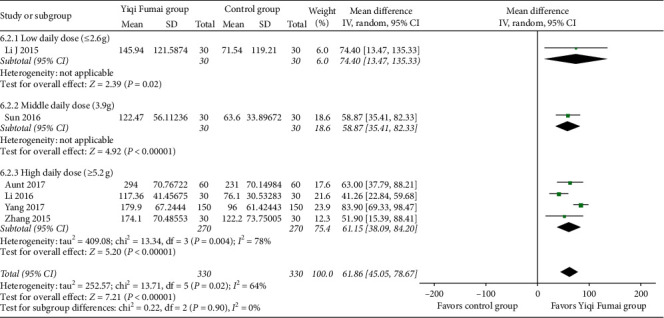
Meta-analysis of the 6-minute walk test performance m.

Six RCTs (*n* = 660) [15, 20, 21, 27, 37, 41] reported changes in the patients' exercise capacity as assessed by the 6MWT. The patients in the YQFM group displayed significantly greater improvement in the 6MWT than those in the control group (MD 61.86 m, 95% CI 45.05 to 78.67, *p* < 0.00001; Figure 7). The heterogeneity was moderate (*I*^2^ = 64%).

#### 3.4.7. QoL

**Figure 8:**
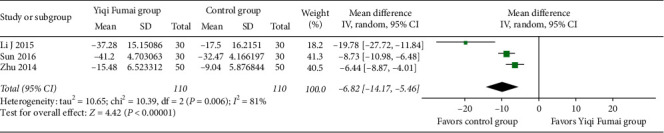
Meta-analysis of the Minnesota Living with Heart Failure questionnaire.

Three RCTs (*n* = 220) [21, 27, 43] assessed the QoL using the Minnesota Living with Heart Failure questionnaire. After the treatments, the magnitude of reduction (improvement) in the questionnaire scores in the YQFM group was significantly greater than that in the control group (MD −9.82, 95% CI −14.17 to −5.46, *p* < 0.00001; *I*^2^ = 81%; Figure 8).

### 3.5. Subgroup Analysis

The subgroup analyses showed a statistically significant increased tendency in the LVEF along with an increase in the YQFM daily dose (low vs. medium vs. high: MD 3.92% vs. 5.18% vs. 6.09%, interaction *p*=0.03; Figure 2), a significantly higher increase in cardiac output in patients with an average age <60 years (<60 versus ≥60 years: 0.51 L/min vs. 0.26 L/min, interaction *p*=0.003), and a significant higher improvement in the 6MWT in patients with an average age <60 years (<60 versus ≥60 years: 0.51 L/min vs 0.26 L/min, interaction *p*=0.03) and in RCTs with a moderate overall risk of bias (moderate vs. high: MD 83.90 m vs. 52.56 m, interaction *p*=0.0010). The other subgroup analyses showed no significant subgroup differences (all interactions *p* > 0.05). Subgroup analyses of the LVESD and QoL were not performed because there were too few RCTs. The details of the subgroup analyses are shown in Tables S1–S3 in the supplementary files.

### 3.6. Safety

Thirteen [12, 22–24, 26, 28–30, 33, 37, 40–42] of the 35 RCTs reported AEs during treatment, including 11 RCTs that reported no AEs in either group and two RCTs [30, 42] that reported AEs in detail. One RCT [30] reported that 0 (0.0%), 2 (3.39%), 1 (1.69%), and 1 (1.69%) patients in the YQFM group and 4 (6.78%), 2 (3.39%), 2 (3.39%), and 4 (6.78%) patients in the control group had gastrointestinal reactions, nausea, vomiting, and upper abdominal discomfort, respectively, and the incidence of these AEs did not significantly differ between the two groups. Whether these AEs were associated with YQFM was uncertain. Another RCT [42] reported one (10%) case of dry mouth in the control group and one (10%) case of nausea and vomiting in the YQFM group. The correlation between nausea or vomiting and YQFM was also not reported.

### 3.7. Publication Bias

According to the funnel plots (Figures S3–S5 in supplementary files), the effect estimates of the response to treatment, LVEF, and NT-proBNP were distributed symmetrically, indicating that the possibility of publication bias was small. This finding is consistent with the results of Egger's tests (response to treatment: *p*=0.687; LVEF: *p*=0.527; NT-proBNP: *p*=0.062) and Begg's tests (response to treatment: *p*=0.533; LVEF: *p*=0.355; NT-proBNP: *p*=0.063). The other outcomes were not tested because of insufficient data.

## 4. Discussion

To the best of our knowledge, this study is the first systematic review to assess YQFM for CHF. The findings showed that as a complementary therapy, YQFM combined with conventional treatment presented a remarkably improved efficacy in the primary outcome compared with the control group as the YQFM group had a 12.8% higher probability of having a marked response rate and an 88% higher probability of improving by one class in response to treatment. YQFM also significantly improved all secondary outcomes, including the LVEF, cardiac output, LVESD, NT-proBNP, 6MWT, and QoL, in CHF patients. There was a dose-response effect on the LVEF in which a high YQFM dose provided significantly better improvement than a low dose.

The response to treatment is based on an evaluation of the NYHA cardiac function classification, which mainly reflects cardiac function in terms of the intensity of the physical activity that patients can perform. Therefore, the effects of YQFM on the response to treatment can be verified by the results of another physical test, i.e., the 6MWT, and both tests ultimately reveal significant improvements in the QoL. YQFM also increased the LVEF, a percentage of stroke volume of the end-diastolic volume of the ventricle that is positively associated with cardiac output and inversely associated with the LVESD; these outcomes can all predict the prognosis of CHF patients. Based on the effect size, 12.8% of the patients eventually achieved class I heart function or improvement in heart function by ≥ 2 classes and a 5.15% increase in the LVEF, and these results are even better than the average efficacy of Western medicines [44]; thus, we believe that the additional improvements in the cardiac function indices provided by YQFM have a promising clinical value. The NT-proBNP level is also an independent factor predicting a poor prognosis of CHF, such as all-cause death and cardiovascular disease/heart failure hospitalization. Although the cut-off value used to evaluate prognosis is not completely clear, a decrease of -341.83 pg/mL can be considered meaningful for improving the prognosis of CHF patients. Overall, YQFM is clinically effective in treating CHF patients based on the findings of this systematic review. According to the results of the subgroup analysis, YQFM is effective for CHF patients of all ages, and the daily dose of YQFM could be increased to 5.2 g/day to ensure safety and further improve the efficacy.

A few studies have investigated the mechanism of the entire formula of YQFM in the treatment of CHF. Therefore, we attempt to discuss the underlying mechanism of each component of YQFM. Ophiopogonin, the active component of Radix Ophiopogonis, is the largest component (54.5%) of YQFM; it has been demonstrated to be effective in inhibiting endoplasmic reticulum stress and reducing myocardial cell apoptosis by upregulating cytochrome P450 2J2 and epoxyeicosatrienoic acids, which can further inhibit myocardial hypertrophy, stabilize myocardial cell membrane, and improve positive inotropic action [45]. An important cause of CHF is myocardial ischaemia/reperfusion injury, which leads to the highly sensitive state of mitochondrial permeability caused by Ca^2+^ stimulation. Schizandrol, the second active component (27.3%), can reduce mitochondrial sensitivity; it therefore, plays a role in protection against myocardial ischaemia/reperfusion injury and has cardiovascular protection effects such as controlling the ventricular rate, anti-inflammation, and anti-cell apoptosis [46]. Total saponins panax ginseng, the third ingredient (18.2%) of YQFM, can inhibit myocardial cell apoptosis by downregulating caspases in apoptotic signal transduction, improving myocardial remodelling caused by CHF, enhancing myocardial contractility, and finally, recovering myocardium damage after ischaemia and hypoxia [47]. Although all single components have been proven to be beneficial for CHF, the abovementioned mechanism evidence was all based on animal experiments. The specific mechanism in humans and whether the three TCMs have synergistic effects remain unclear.

YQFM appears to be relatively safe because only two of the 33 included RCTs reported mild gastrointestinal side effects in the YQFM group. However, observational safety monitoring data show that YQFM is not completely safe. For instance, a retrospective study [48] of 2476 patients receiving YQFM reported 31 AEs after YQFM (incidence: 1.25%), including 26 common AEs and five serious AEs; most of the AEs were systemic damages and allergic reactions on the skin, and all of them were remitted after treatment. Another study [49] summarized 42 AEs after YQFM treatment from 2007 to 2014; of these AEs, palpitations (16.3%), drowsiness (10.2%), and rashes (10.2%) were the most common. However, the degree of the association between YQFM and AEs was unclear in both studies. In any case, the clinical use of YQFM requires careful monitoring of adverse reactions and prompt treatment. The drip rate of YQFM is recommended to be lower than 40 drops per minute based on the results of a study investigating the risk factors of adverse reactions to YQFM [50].

This study has some limitations. First, due to methodological flaws such as inappropriate randomization methods and the lack of reporting allocation concealment and blinding methods, the included RCTs had an overall moderate-to-high risk of bias. Second, the residual heterogeneity in the LVEF, NT-proBNP, and QoL remained high after the explanation of the subgroup analyses, which may impact the accuracy of the effect estimates. We cannot perform subgroup analyses of the two suspected sources of heterogeneity, different baseline NYHA cardiac functions and different types of cointerventions, since the subgroup data were not separately reported in the primary studies. Nevertheless, regarding these outcomes, most results of individual RCTs are positive, and the negative individual results were generally marginally significant and/or had a small weight; thus, we believe that the high heterogeneity is unlikely to distort the direction of the results favouring the YQFM group. The meta-analytic estimates averaged by a random-effects model are still clinically meaningful. Third, because all included RCTs had short follow-up periods, this study was unable to evaluate the effects of YQFM on long-term CHF endpoints (e.g., mortality).

## 5. Conclusions

Our findings suggest that YQFM is an effective complementary treatment for CHF that can significantly improve the response to treatment, LVEF, cardiac output, LVESD, NT-proBNP, 6MWT, and QoL. YQFM dose-dependently improves the LVEF. Although the RCT evidence shows that YQFM is relatively safe, data from retrospective studies suggest that the clinical use of YQFM still needs careful safety monitoring. Due to the limitations of the risk of bias and heterogeneity, more high-quality studies are necessary to further verify the efficacy and safety of YQFM for CHF.

## Figures and Tables

**Table 1:** Baseline characteristics of included studies.

Study	Sample size (T/C)	Male (T/C)	Mean age (T/C)	Cardiac function (T/C)			Interventions in both Yiqi Fumai injection and control group	Dose of Yiqi Fumai injection	Treatment period (weeks)	Outcomes
				NYHA II	NYHA III	NYHA IV				
Aunt and Nurbia [15]	60/60	—	55.4 (total)	—	—	—	Furosemide, metoprolol	5.2 g	2	a, b, f
Diao [16]	43/43	22/21	60.3/60.5	—	—	—	Spironolactone, digoxin	—	4	b
Dong and Liu [17]	60/60	38/35	56.4/56.1	—	—	—	Enalapril, metoprolol	5.2 g	2	a, b, e
Feng [18]	27/24	—	56.0∼86.0	12/10	12/12	3/2	Spironolactone, metoprolol	5.2 g	—	b, d
Li [19]	30/30	14/17	67.5/64.7	16/18	14/12	—	Furosemide, metoprolol	1.3 g	2	b
Li et al. [20]	30/30	15/15	71.1/70.9	—	—	—	Ramipril, furosemide	5.2 g	1.5	e, f
Li [21]	30/30	—	64.3 (total)	—	—	—	Furosemide, captopril	2.6 g	4	b, f, g
Li et al. [22]	59/59	35/34	68.5/68.3	—	—	—	Atorvastatin	5.2 g	2	b, e, h
Liu [23]	45/45	24/25	56.7/55.2	/	19/18	26/27	Dobutamine hydrochloride	5.2 g	2	b, e, d, h
Lv [24]	43/40	30/28	62.7/61.5	17/16	14/13	12/11	Furosemide, metoprolol	2.6 g	2	h
Mao and Song [25]	30/30	17/18	58.5/58.6	—	—	—	Spironolactone, metoprolol	2.6 g	2	b, e
Ren [26]	44/44	—	55.0 (total)	—	—	—	Trimetazidine	5.2 g	2	b, e, h
Sun [27]	30/30	11/10	76.0/74.0	6/5	15/17	9/8	Enalapril, metoprolol	3.9 g	2	a, b, e, f, *c*, g
Sun [28]	60/60	43/44	58.4/58.2	16/17	26/27	18/16	Captopril, metoprolol	5.2 g	2	b, e, h
Wang and He [29]	35/29	19/16	54.9/52.5	9/7	20/18	6/4	Furosemide, digoxin	3.9 g	2	b, c, d, h
Wang [30]	50/50	32/30	70.0/69.5	—	—	—	Aspirin, spironolactone	5.2 g	2	b, e, h
Wang et al. [31]	33/32	19/18	64.3/63.8	—	—	—	Milrinone	5.2 g	1	a, b, e, c
Wang [32]	49/49	32/33	76.0/79.0	—	—	—	Metoprololsuccinate	5.2 g	2	b
Wang and Niu [33]	35/35	21/22	58.0/59.0	10/10	18/19	7/6	Isosorbide dinitrate	3.9 g	2	b, c, h
Wu [34]	32/30	19/17	60.3/62.4	—	—	—	Enalapril	2.1 g	2	b, c
Xi [35]	79/79	39/36	56.0/56.0	—	—	—	Amiodarone, furosemide	2.6 g	2	a
Xue et al. [13]	44/43	28/25	55.3/55.5	7/6	28/28	9/9	Captopril, metoprolol	2.6 g	2	a, b
Yang and Liu [36]	60/60	—	72.2/73.1	6/8	54/52	—	Enalapril, metoprolol	5.8 g	2	b, e
Yang [37]	150/150	89/87	57.2/56.4	29/28	96/97	25/25	Captopril, digoxin	5.2 g	2	b, e, f, h
Yang et al. [38]	30/30	22/23	70.7/70.8	8/5	18/18	4/7	Enalapril, metoprolol	5.2 g	2	b, e
Yang [39]	30/30	16/15	69.0/67.8	6/6	16/18	8/7	Furosemide, metoprolol	2.6 g	2	a
Yu and Wang [40]	30/30	15/16	66.0/65.0	6/6	21/20	3/4	Furosemide, spironolactone	3.9 g	2	b, h
Yu et al. [11]	45/45	25/23	51.5/52.0	—	—	—	Spironolactone, metoprolol	2.6 g	2	a, b
Zhai and Hui [12]	30/30	—	63.0 (total)	20	36	4	Ramipril, furosemide	5.2 g	2	a, b, e, h
Zhang [41]	30/30	—	70.0 (total)	26	20	14	Enalapril, metoprolol	5.2 g	2	a, b, e, f, h
Zhang [10]	40/40	23/24	56.3/55.9	—	—	—	Furosemide, captopril	3.9 g	2	a, b, e
Zhu [42]	100/100	66/66	63.7/63.2	—	—	—	Atorvastatin	5.2 g	2	b, d, h
Zhu and Han [43]	50/50	31/28	66.2/69.0	—	—	—	Aspirin, spironolactone	5.2 g	2	a, b, *c*, d, g

*Notes*. Both groups received the same basic treatment; “—” = not reported; T = trial group; C = control group; NYHA = New York Heart Association. Outcomes: a = response rate; b = left ventricular ejection fraction; c = cardiac output; d = left ventricular end-systolic diameter; e = N-terminal pro-brain natriuretic peptide; f = 6-minute walk test; g = quality of life; h = adverse reaction.

## Data Availability

The datasets used and/or analyzed during the current study are available from the corresponding author on reasonable request.

## Abbreviations

CI: Confidence interval
MD: Mean difference
pOR: Proportional odds ratio
RCT: Randomized controlled trial
CHF: Chronic heart failure
YQFM: Yiqi Fumai injection
NYHA: New York Heart Association
LVEF: Left ventricular ejection fraction
LVESD: Left ventricular end-systolic diameter
NT-proBNP: N-terminal pro-brain natriuretic peptide
6MWT: 6-minute walk test
QoL: Quality of life
TCM: Traditional Chinese medicine
AEs: Adverse events.

## Supplementary Materials

Supplementary Materials Figures S1-S2 : results of the risk of bias assessment. Figures S3–S5 : funnel plots. Table S1–S3 : results of the subgroup analyses.
