# Supplementary material for: Yiqi Fumai Injection as an Adjuvant Therapy in Treating Chronic Heart Failure: A Meta-Analysis of 33 Randomized Controlled Trials
Source: Evid Based Complement Alternat Med. 2020 Aug 19;2020:1876080. doi: 10.1155/2020/1876080 (PMC7453275; doi:10.1155/2020/1876080)
Supplement: Supplementary Materials — Figures S1-S2 : results of the risk of bias assessment. Figures S3–S5 : funnel plots. Table S1–S3 : results of the subgroup analyses. [file 1876080.f1.pdf]

Supplementary files

Figures S1-S2: Results of the risk of bias assessment.

Figures S3-S5: Funnel plots.

Table S1-S3: Results of the subgroup analyses.

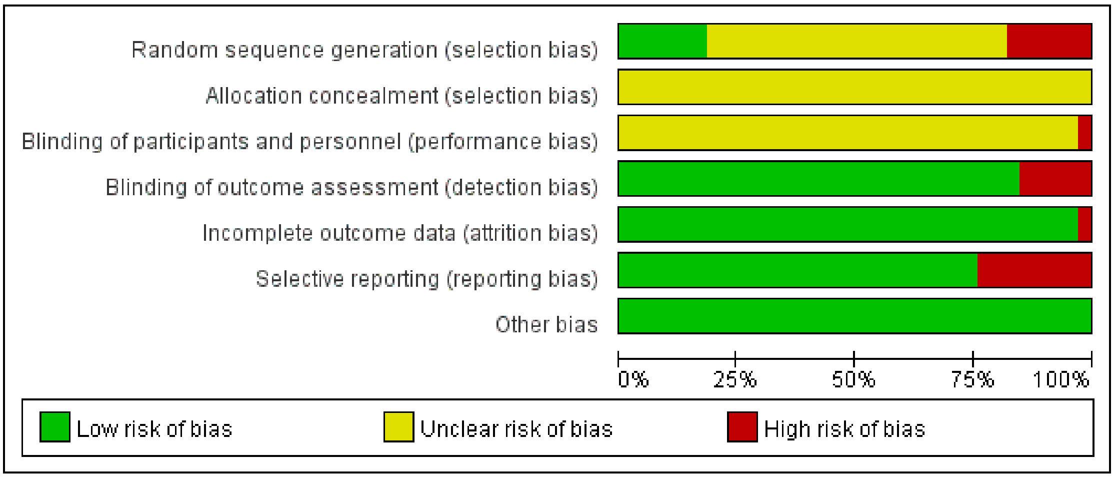

Figure S1. Percentage of the risk of bias

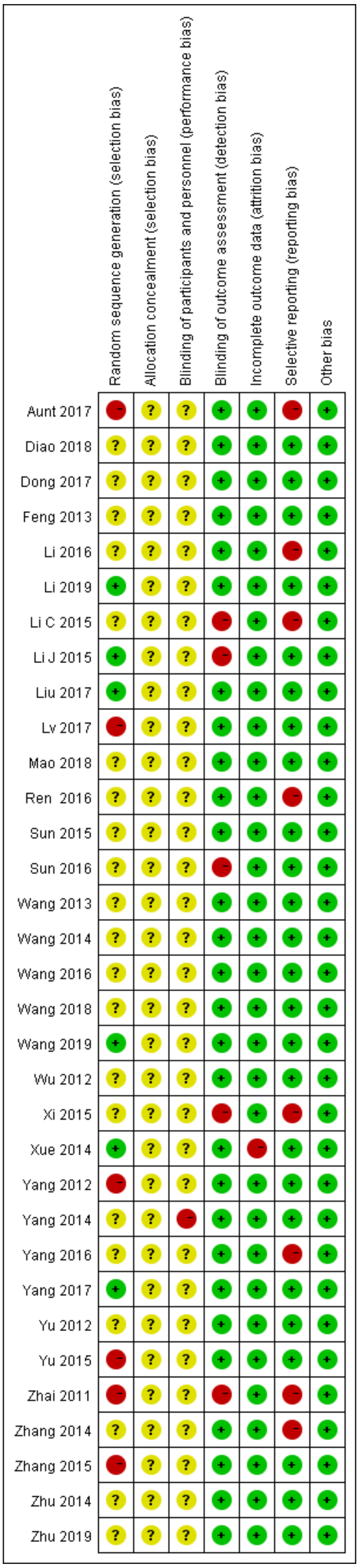

Figure S2. Risk of bias assessment of the included studies.

Note: "+" indicates low, "-" indicates high, and "?" indicates an unclear risk of bias.

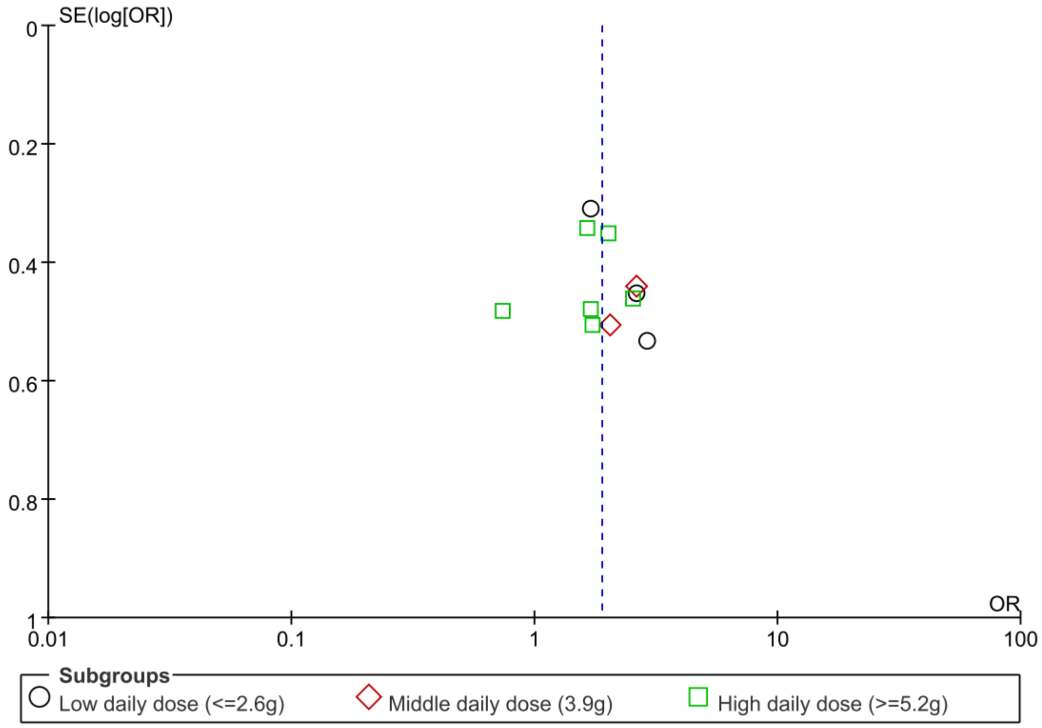

Figure S3. Publication bias funnel plots of the response to treatment.

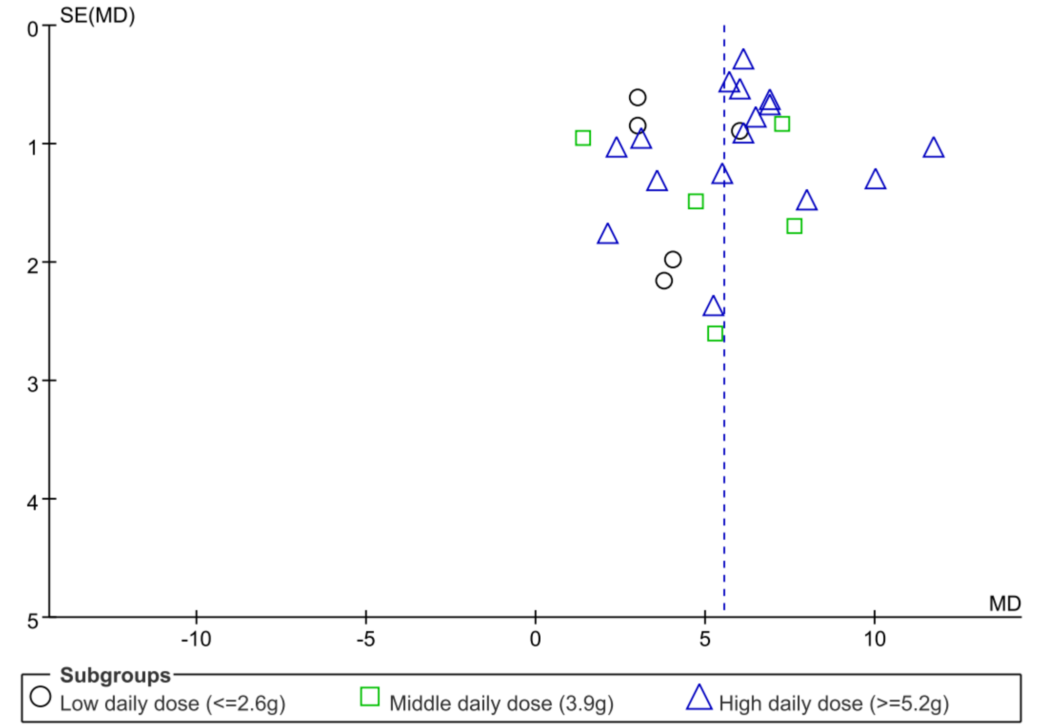

Figure S4. Publication bias funnel plots of the left ventricular ejection fraction.

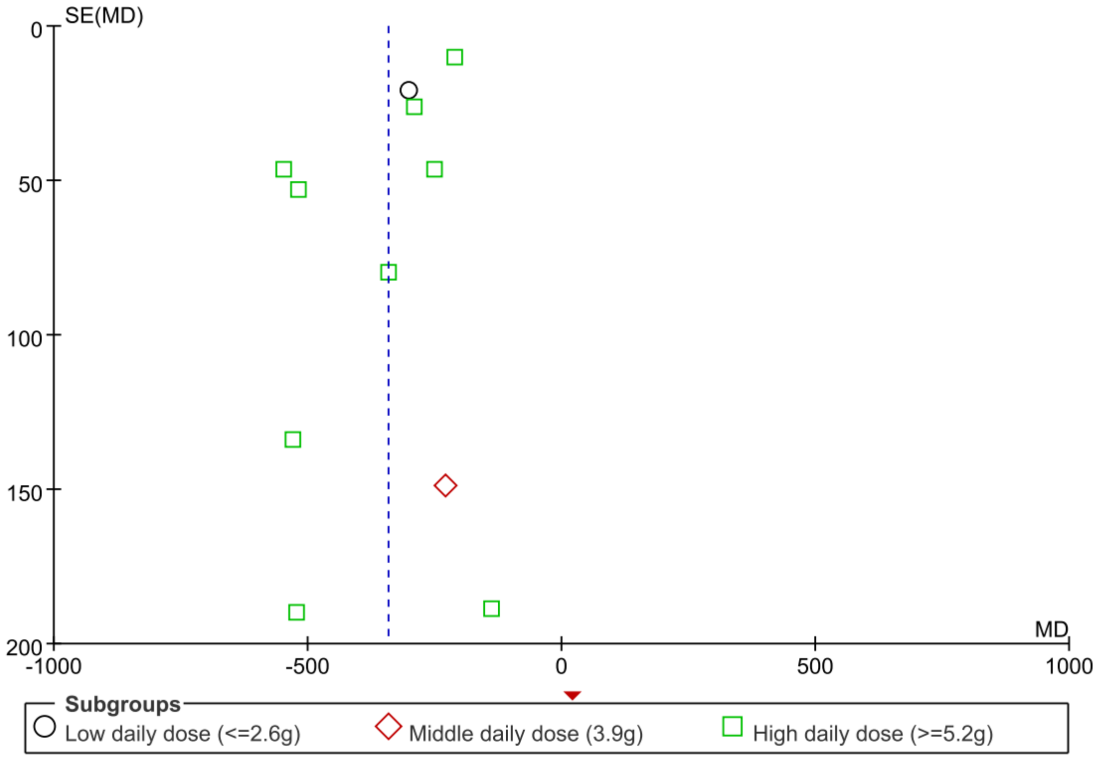

Figure S5. Publication bias funnel plots of N-terminal pro-brain natriuretic peptide.

Table S1. Results of the subgroup analysis stratified by the daily dose of Yiqi Fumai injection.

Outcomes	Number of studies	Low daily dose (≤2.6 g)		Middle daily dose (3.9 g)		High daily dose (≥5.2 g)		Interaction P
		OR/MD (95% CI)	I^2	OR/MD (95% CI)	I^2	OR/MD (95% CI)	I^2	
Response to treatment	11	2.09 (1.33 to 3.29)	0%	2.34 (1.22 to 4.50)	0%	1.68 (1.20 to 2.35)	0%	0.57
LVEF (%)	26	3.92 (2.56 to 5.28)	52%	5.18 (2.33 to 8.02)	83%	6.09 (5.20 to 6.98)	80%	0.03
Cardiac output (L/min)	6	0.40 (-0.70 to 1.50)	NA	0.37 (0.15 to 0.59)	77%	0.22 (0.03 to 0.41)	0%	0.60
NT-proBNP (pg/ml)	14	-301.00 (-342.28 to -259.72)	NA	-157.93 (-406.03 to 90.18)	0%	-369.06 (-469.49 to -268.64)	90%	0.23
6MWT (m)	6	74.40 (13.47 to 135.33)	NA	58.87 (35.41 to 82.33)	NA	61.15 (38.09 to 84.20)	78%	0.90

Notes: OR = odds ratio; MD = mean difference; CI = confidence interval; LVEF = left ventricular ejection fraction; NT-proBNP = N-terminal pro-brain natriuretic peptide; NA = not applicable

Table S2. Results of the subgroup analysis stratified by the patients' average age.

Outcomes	Number of studies	<60 years		≥60 years		Interaction P
		OR/MD (95% CI)	I^2	OR/MD (95% CI)	I^2	
Response to treatment	11	1.97 (1.42 to 2.71)	0%	1.77 (1.19 to 2.62)	0%	0.68
LVEF (%)	25	6.28 (4.29 to 8.26)	89%	5.20 (4.42 to 5.98)	73%	0.32
Cardiac output (L/min)	6	0.51 (0.35 to 0.66)	0%	0.26 (0.19 to 0.33)	0%	0.003
NT-proBNP (pg/ml)	14	-232.03 (-374.96 to -89.09)	99%	-334.80 (-566.74 to -102.85)	99%	0.46
6MWT (m)	6	76.09 (56.27 to 95.91)	49%	49.72 (36.57 to 62.87)	0%	0.03

Notes: OR = odds ratio; MD = mean difference; CI = confidence interval; LVEF = left ventricular ejection fraction; NT-proBNP = N-terminal pro-brain natriuretic peptide; NA = not applicable

Table S3. Results of the subgroup analysis stratified by the level of risk of bias.

Outcomes	Number of studies	Moderate risk of bias		High risk of bias		Interaction P
		OR/MD (95% CI)	I^2	OR/MD (95% CI)	I^2	
Response to treatment	11	1.87 (1.17 to 2.98)	0%	1.89 (1.41 to 2.54)	0%	0.96
LVEF (%)	26	5.22 (4.36 to 6.09)	75%	5.98 (4.26 to 7.70)	88%	0.44
Cardiac output (L/min)	6	0.36 (0.19 to 0.53)	31%	0.26 (0.19 to 0.33)	NA	0.29
NT-proBNP (pg/ml)	14	-200.37 (-357.64 to -43.10)	99%	-307.60 (-470.93 to -144.27)	97%	0.35
6MWT (m)	6	83.90 (69.33 to 98.47)	NA	52.56 (40.90 to 64.22)	64%	0.0010

Notes: OR = odds ratio; MD = mean difference; CI = confidence interval; LVEF = left ventricular ejection fraction; NT-proBNP = N-terminal pro-brain natriuretic peptide; NA = not applicable
